# Total collected dialysate lithium concentration after successful dialysis treatment in case of intoxication

**DOI:** 10.1186/2050-6511-15-49

**Published:** 2014-09-06

**Authors:** Julius J Schmidt, Johan Lorenzen, Christos Chatzikyrkou, Ralf Lichtinghagen, Jan T Kielstein

**Affiliations:** 1 Department of Nephrology and Hypertension, Medical School Hannover, Carl-Neuberg-Strasse 1, 30625 Hannover, Germany; 2 Institute of Clinical Chemistry, Medical School Hannover, Hannover, Germany

**Keywords:** Lithium, Intoxication, Intensive care unit, Drug monitoring, Extended dialysis

## Abstract

**Background:**

Lithium intoxication has potentially fatal neurologic and cardiac side effects. Extracorporeal removal can therefore be lifesaving. The dialysance of lithium is high as it is a small molecule. Comparable to its neighbor in the periodic table, sodium, its intracellular accumulation hampers its removal by renal replacement therapy, despite its favorable size. For this reason the combination of short intermittent and prolonged dialysis may be a beneficial approach in acute lithium intoxication, yet only a report of such a combination has been published and actual removed lithium has not been quantified.

**Case presentation:**

We describe the first measurement of lithium in the spent total dialysate treating an acute lithium overdose of a 44 year old Caucasian patient on chronic lithium therapy, undergoing extended dialysis. Extracorporeal therapy was initiated at a lithium serum concentration of 3.24 mmol/l. With blood/dialysate flow of 350 ml/min the 1.3 m^2^ polysulfone dialyzer exhibited a maximum lithium clearance of 177 ml/min. After 4.1 hours of treatment the lithium level was lowered to 1.25 mmol/l. In the total spent dialysate 250 mg lithium, i.e. ~ 40% of the ingested amount were found. The subsequent extended dialysis over 9.5 hours further decreased serum levels to 0.79 mmol/l. Neurological symptoms improved within the first 60 min of treatment. The patient could be transferred to a psychiatric hospital on the morning after admission.

**Conclusion:**

Standard intermittent hemodialysis with subsequent extended dialysis can efficiently be employed in severe lithium intoxication by combining prompt a fast decrease of lithium blood levels and preventing rebound/assuring removal of redistributed lithium.

## Background

Lithium is a commonly used drug in psychiatric therapy since 1949 [1]. It is prescribed for the treatment of several psychiatric diseases including chronic therapy of bipolar disorder [2]. In 1929 Lithium was even an ingredient in the soft drink “Bib-Label Lithiated Lemon-Lime Soda”, which later became better known as “7up” [3].

Although inexpensive, its narrow therapeutic range and the advent of newer drugs for which therapeutic drug monitoring is not necessary have led to a decrease in Lithium prescription. Although lithium reduces the risk of suicide in patients with mood disorders [4], the drug can also be used in suicidal intention. Lithium intoxication remains one of the top five toxins for which extracorporeal therapies are used [5]. Serum levels of 1.5 μmol/l can already cause intoxication symptoms like somnolence, arrhythmia or polyuria due to a renal diabetes insipidus [6]. The recommended therapeutic lithium plasma level is 0.6-1.5 mmol/l. After oral administration the bioavailability is >95% and there is no relevant protein binding of lithium. Lithium is renally excreted with an elimination half-life of 24 hours. The half life may increase to as much as 60 hours in chronic lithium therapy. Lithium is a very small molecule with a molecular weight of 7 Dalton. Due to its small size and sodium like characteristics it can easily be removed by hemodialysis [6].

Extended dialysis is a mostly diffusive dialysis modality with a prolonged treatment duration of 8–12 hours. By using the GENIUS batch dialysis system, the total spent dialysate can be measured after each treatment. Structure and function of the Genius 90 l dialysis system as well as sample collection are described elsewhere [7]. Even though dialysis is a well-described treatment for lithium intoxications [8] there is scarce data about the elimination of lithium in extended dialysis. To our knowledge, this is the first case report in which both, lithium clearance and the total amount of eliminated lithium in the total spent dialysate have been measured, allowing that the change in plasma levels can be related to the total removal of lithium.

## Case presentation

A 44 year old Caucasian female (weight: 65 kg height: 170 cm) with a history of bipolar disorder and Wolf-Parkinson-Withe syndrome was transferred from a local hospital to our intensive care unit due to somnolence with a GCS (Glasgow Coma Scale) of 9 and polyuria after acute lithium intoxication. The patient was treated with a lithium medication for years. She took 4 tablets of perazindimalonat, 29 tablets of lithium (0.022 g Lithium per pill) and 10 tablets of zolpidemtartrat in suicidal attempt. On admission to our emergency room the patient’s history could not be obtained due to somnolence. Patient’s heart rate was 60 bpm, blood pressure was 120/80 mmHg, respiratory rate 14 per minute, cardiac and pulmonary auscultation were unremarkable as was the remainder of the physical exam. The patient was polyuric (4 l urine in 24 hours). Laboratory work-up showed a serum creatinine of 57 μmol/l, a serum sodium of 140 mmol/l and a serum potassium of 4.0 mmol/l. Liver function test, blood gas analysis and whole blood count were normal. Lithium serum level on admission was 3.9 mmol/l. Subsequently it rose to a concentration of 4.2 mmol/l two hours after admission. Due to the combination of the rising lithium serum level and the neurological symptoms the patient was transferred to the ICU for monitoring and the critical care nephrology department was consulted.

For fast reduction of lithium levels an intermittent hemodialysis using the GENIUS dialysis batch system (FMC, Germany) with a 90 liters dialysate tank volume and a 1.3 m^2^ F60S polysulfone high-flux dialyzer (FMC, Germany) was started. Blood and dialysate flow rate was started at 350 ml/min. As the patient woke up and became more vigilant but also agitated after one dialysis treatment, the flow rate had to be reduced to 200 ml/min to avoid frequent system alarms and the need to restrain the patient. Ultrafiltration rate was adjusted at minimum rate of 50 ml/h. Anticoagulation was provided by unfractionated heparin medication with an initial bolus of 1000 IE and a continuous dose of 500 IE per hour. Lithium serum levels were measured right before the start of the treatment, as well as within an interval of 15 minutes, 30 minutes, 1 hour, 2 hours and 3.5 hours after treatment initiation. One of the peculiarities of the GENIUS dialysis system is that fresh and spent dialysate are separated by virtue of temperature differences and difference in uremic solute concentration [9]. To prevent an early contamination of the fresh with the spent dialysate as seen in GENIUS therapy in patients without renal failure [10], dialysis therapy was stopped after 4 hours of treatment. Plasma dialyzer clearance rates were measured 30 minutes and 3 hours after dialysis initiation using established dialyzer clearance equations reported previously [11]. Lithium dialyzer clearance was calculated according to the following equation: Kplasma = QB × (1 – Hct/100) × ((Cart – Cven)/Cart). Pre dialyzer concentrations (Cart) and post dialyzer concentration (Cven) were drawn at a time of minimal ultrafiltration (50 ml/h).

**Figure 1:**
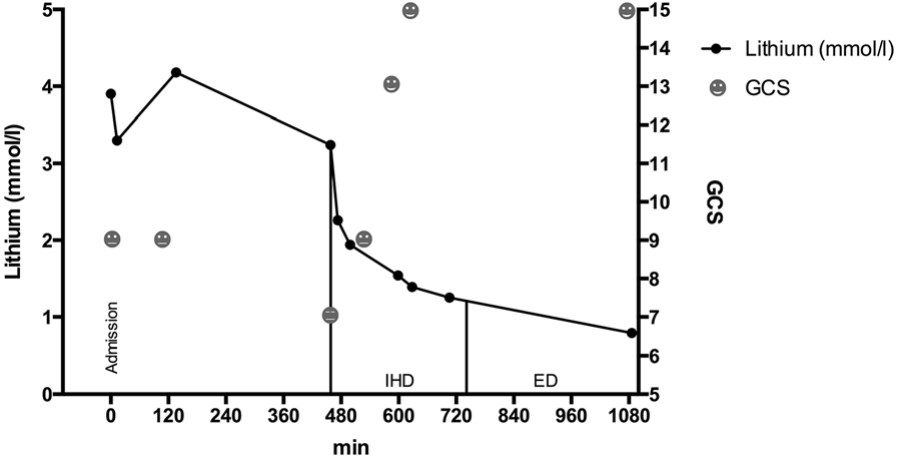
**Time course of serum lithium concentration over the time of ICU stay and corresponding GCS scores of the patient.** GCS = Glasgow Coma Scale, IHD = intermittent hemodialysis, ED = extended dialysis.

After the marked clinical improvement already occruing after the first hour of intermittent hemodialysis a subsequent extended dialysis with dialysate and blood flow of 140 ml/min was initiated right after the completion of the first dialysis session using a second GENIUS machine. As there was no gap between treatments a rebound could not be assessed.During the first dialysis lithium serum level was lowered by 71.5%. Plasma dialyzer clearance rate was 177.4 ml/min after 30 minutes of dialysis at a blood flow of 350 ml/min and 129.6 ml/min after 3 hours of dialysis at a blood flow of 260 ml/min. We mixed the total spent dialysate of the first dialysis tank by air insufflation and collected a sample for laboratory measurement. Lithium dialysate level was 0.4 mmol/l in the dialysate tank of 90 liters volume. Accordingly we found a total amount of 250 mg of lithium in the total spent dialysate. 5.5 hours after the initiation of the second dialysis therapy serum lithium level was 0.79 mmol/l (Figure 1). No neurological symptoms were detectable after the patient woke up fully. Blood pressure rose to 175/85 mmHg prompting treatment with urapidil. Crystalloids were administered to compensate for polyuria. The morning after admission, the patient had fully recovered. Intensive monitoring was no longer required and the patient was retransferred to the psychiatric hospital. Blood pressure rose meanwhile up to 175/85 mmHg and urapidil therapy was initiated. Crystalloid solutes were administered to provide adequate fluid supply during polyuria. The morning after admission, the patient had fully recovered. Monitoring was no longer required and the patient was retransferred to the psychiatric hospital.

## Conclusion

Hemodialysis is a well-known technique for lithium elimination in case of lithium intoxication. However, data about the elimination of lithium by extended dialysis are scarce. To our knowledge this is the first report providing dialysate data on lithium elimination in the total spent dialysate. In this case we achieved a lithium serum level reduction of 71.5% by a total elimination of only 250 mg of lithium during a 4 h dialysis treatment. The patient supposedly took a dose of about 638 mg of lithium. As volume of distribution values of 0.9 l/kg are reported in the literature it can be hypothesized, the patient either took a lower dose of lithium or the ingested pills had not been completely absorbed. However, additional urine lithium excretion was not measured.

Lithium has a 4 fold higher intracellular than extracellular concentration and its equilibration between intracellular and extracellular space needs transport via sodium transport proteins. Extrusion of lithium is even prolonged in chronic lithium therapy [6]. So prolonged dialysis seems beneficial to remove lithium under a constant cellular shift from intracellular to extracellular space after acute intoxication [8]. Previously CVVHDF was successfully used to prevent lithium rebound after lithium serum level reduction via hemodialysis [12].

Hemodialysis is generally preferred for lithium elimination [13]. Continuous hemofiltration for 24 to 36 hours may also be prescribed for this purpose [14], if much longer treatment durations are needed for an effective removal of lithium. In our case the patient was transferred already 20 hours after admission.

As seen in many studies before, lithium clearance ranges between 70 to 170 ml/min highly depending on the prescribed blood flow during intermitted hemodialysis [13]. These findings match with our results. We measured a dialyzer clearance of 177.4 ml/min using a blood flow of 350 ml/min and a dialyzer clearance of 129.6 ml/min running a blood flow of 260 ml/min. The blood flow of 350 ml/min is the maximum rate of the GENIUS dialysis system. Higher blood flow rates may lead to even higher clearance rates, but their clinical relevance may be limited. However, blood flow adjustment may regulate the lithium excretion efficacy and dialysis therapy can be adapted according to therapeutic needs. Additionally, routine serum measurements like sodium may help predicting the required amount of dialysis in severe lithium intoxication [15].

Extended dialysis has been successfully performed in the past to reduce lithium serum levels after acute intoxication without a major rebound phenomenon [16], however the total eliminated amount of lithium has not been measured so far. The rather low total amount of lithium (in relation to the allegedly ingested amount) that has been eliminated during the first 4 hours of dialysis suggests that prolonged dialysis like extended dialysis is advisable to augment treatment of acute lithium intoxication to minimize the risk of a potentially dangerous lithium rebound. The data of a single case along with the limitations pointed out in the manuscript do not allow suggesting that one modality of renal replacement therapy is superior to another yet extended dialysis after an initial intermittent hemodialysis seems to be a prudent approach to intoxicated patients.

## Consent

Written informed consent was obtained from the patient for publication of this Case report and any accompanying images. A copy of the written consent is available for review by the Editor of this journal.

## Competing interests

The authors declare that they have no competing interests.

## Authors’ contributions

JJS, JL, CC and JTK were the treating physicians of the patient reported. RL provided laboratory measurements. JJS and JTK evaluated the test results. All of the authors have participated in the discussion and in writing of the submitted manuscript. All authors read and approved the final manuscript.

## Pre-publication history

The pre-publication history for this paper can be accessed here:

http://www.biomedcentral.com/2050-6511/15/49/prepub
